# Is there a link between appendicitis and the risk of diverticular disease?: a large German cohort study

**DOI:** 10.1007/s00384-024-04624-9

**Published:** 2024-04-10

**Authors:** Sarah Krieg, Ernst W. Kolbe, Sven H. Loosen, Christoph Roderburg, Andreas Krieg, Karel Kostev

**Affiliations:** 1 Department of Inclusive Medicine, University Hospital Ostwestfalen-Lippe, Bielefeld University, 33617 Bielefeld, Germany; 2 Department of General and Visceral Surgery, Thoracic Surgery and Proctology, Medical Campus OWL, University Hospital Herford, Ruhr University Bochum, 32049 Herford, Germany; 3 Department of Gastroenterology, Hepatology and Infectious Diseases, Medical Faculty, University Hospital Duesseldorf, Heinrich Heine University Duesseldorf, 40225 Duesseldorf, Germany; 4 IQVIA, 60549 Frankfurt, Germany

**Keywords:** Appendicitis, Appendectomy, Diverticular disease, Gut microbiota, Epidemiology, Risk

## Abstract

**Purpose:**

Appendicitis, characterized by inflammation of the vermiform appendix, is a common abdominal emergency necessitating appendectomy. Recent evidence suggests a potential link between appendicitis and subsequent diverticular disease, yet population-based studies investigating this association are limited.

**Methods:**

Utilizing the Disease Analyzer database encompassing data from over 1000 primary care practices in Germany, we conducted a retrospective cohort study. We included 25,379 adults diagnosed with appendicitis and an equal number of matched controls without appendicitis. The incidence of diverticular disease over a 10-year follow-up period was compared between the two cohorts. Cox regression analysis was performed to assess the association between appendicitis and diverticular disease, adjusting for potential confounders.

**Results:**

Our findings revealed a significant association between appendicitis and subsequent diverticular disease (HR: 1.76; 95% CI: 1.57–1.97), with an increased risk observed across all age groups. Notably, this association was stronger in men (HR: 2.00; 95% CI: 1.68–2.37) than in women (HR: 1.58; 95% CI: 1.36–1.84). The cumulative 10-year incidence of diverticular disease was higher in patients with appendicitis (6.5%) compared to those without (3.6%). Additionally, we observed a clear age-dependent increase in the incidence of diverticular disease.

**Conclusion:**

This large-scale population-based study provides valuable insights into the interaction between appendicitis and diverticular disease. The study underscores the need for further research elucidating the underlying mechanisms linking appendicitis to diverticular disease. Probiotics emerge as a potential therapeutic avenue warranting exploration in the management of both conditions. These findings have important implications for clinical practice, highlighting the importance of considering appendicitis as a potential risk factor for diverticular disease, particularly in men. Further investigation is warranted to validate these findings and explore potential therapeutic interventions targeting the shared pathophysiological pathways underlying both conditions.

**Supplementary Information:**

The online version contains supplementary material available at 10.1007/s00384-024-04624-9.

## Introduction

In Western countries, with an incidence of 100 per 100,000 individuals annually and a risk of 7–8% over a lifetime, appendicitis is the most frequent cause of an acute abdomen [1, 2]. It has been defined as inflammation of the vermiform appendix, which is connected to the cecum and is thought to play a physiological role in mucosal immune function and serve as a reservoir of gut microbiota [3, 4]. The etiology of appendicitis is not fully understood. It is assumed that causes such as kinking, scarring, inflammatory swelling of the mucosa or enlargement of the lymphatic tissues, fecal impaction, or foreign bodies can obstruct the outflow from the lumen of the appendix into the colon, resulting in increased pressure in the appendix, reduced blood flow to the intestinal wall, and immigration of bacteria [4, 5]. In contrast to “uncomplicated” appendicitis, which is defined as “simple” inflammation of the vermiform appendix, “complicated” appendicitis is typically accompanied by periappendiceal phlegmon with or without perforation, gangrene, or perityphlitic abscess [6]. In all age groups, the standard treatment for acute appendicitis is appendectomy [2]. It is one of the most frequently undertaken surgeries in Germany, with 93,881 procedures in 2022, and is usually done using minimally invasive approaches [7]. Recently, Andersson and co-workers demonstrated in a large cohort study including 212,963 patients identified from the Swedish Inpatient Register and the National Census who underwent appendectomy before the age of 50 years that appendectomy was associated with a lower risk of ulcerative colitis later in life [8]. In contrast, 2 years later, the same group showed an association between appendectomy and an increased incidence of Crohn’s disease in 212,218 patients with appendectomy before the age of 50 from the same registry [9]. Additional evidence suggesting a possible involvement of the appendix in the immunological balance of the digestive tract comes from the observed association of appendectomy with the higher risk of suffering from severe *Clostridium difficile*–associated colitis [10]. However, these data are somewhat conflicting [11], and it remains unclear whether these findings reflect changes in the human gut microbiome or the removal of an organ hosting lymphoid tissue and thus playing a role in human immune function. Interestingly, recent evidence suggests that appendicitis and/or appendectomy may be positively associated with diverticular disease [12–14]. However, as one of the leading digestive disorders, the pathogenesis of diverticular disease seems to be complex. In particular, genetic and environmental factors such as diet, physical activity, and smoking have been well described as risk factors. In contrast to appendicitis, the treatment of diverticular disease is more complex and stage dependent [15–17]. Notably, the morbidity and mortality rates for sigmoid resection for diverticular disease are approximately 3 times higher than for open appendectomy and nearly 30 times higher than for laparoscopic appendectomy [2, 18]. Since population-based studies on this topic are rare, this study aimed to explore the association between appendicitis and diverticular disease by utilizing a large population-based cohort study in outpatient general practices (GPs) in Germany.

## Material and methods

### Database

We designed a retrospective cohort study using data from the Disease Analyzer database (IQVIA). Disease Analyzer consists of information on prescribed medications, clinical diagnoses, and general medical and demographic data. This information is collected anonymously directly from computerized systems used in primary care and specialty physician offices [19]. IQVIA's Disease Analyzer database, which represents about 3% of all GPs in Germany, uses a sampling methodology that utilizes aggregate statistics of all physicians in Germany released annually by the German Medical Association. Based on these statistics, IQVIA formulates the panel design, which includes four strata: specialist group, federal state, category of community size, and physician age. Earlier research has demonstrated that the panel of German practices represented in the Disease Analyzer database is an accurate reflection of both GPs and specialty practices in Germany [19]. Notably, this database has been previously employed in studies concentrating on gastrointestinal disorders [20, 21].

### Study population

**Fig. 1:**
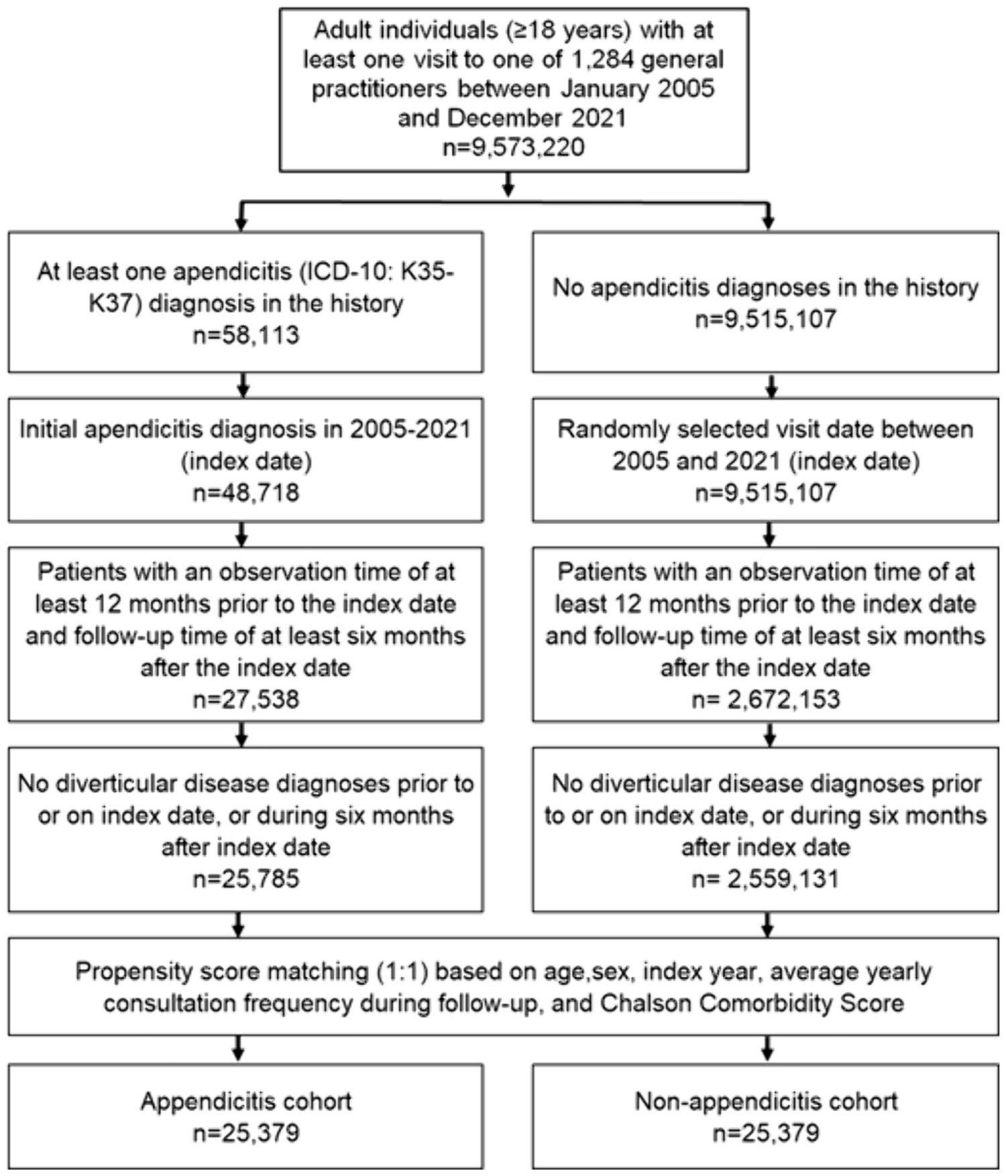
Selection of study patients

Our study included adults (≥ 18 years) with first-time diagnosis of appendicitis (ICD-10: K35-K37) from 1284 GPs in the period January 2005 to December 2021 (index date; Fig. 1) in Germany. Other inclusion criteria included at least 12 months of observation prior to the index date and at least 6 months of follow-up after the index date. Patients diagnosed with diverticular disease (ICD-10: K57) before, on, or within 6 months of the index date were excluded. Individuals not suffering from appendicitis and patients with appendicitis were matched using nearest-neighbor propensity score matching (1:1) after using identical inclusion criteria. Matching parameters comprised age, sex, index year, average number of visits per year during the follow-up period, and the Chalson comorbidity score [22]. The Chalson score is a weight measure that accounts for the number and severity of comorbidities in studies of administrative databases, including a variety of conditions such as macrovascular disease, pulmonary disease, digestive tract disease, liver disease, renal disease, diabetes, AIDS, and others [22]. For the cohort that did not have appendicitis, the index date was defined as a randomly selected visit that occurred in the period January 2005 to December 2021 (Fig. 1).

### Study outcomes and statistical analyses

The outcomes of the study focused on the first diagnosis of diverticular disease within up to 10 years after the index date, taking into account the presence of appendicitis. Differences in sample characteristics and prevalence of diagnoses between both cohorts with and without appendicitis were evaluated by statistical tests: Wilcoxon signed-rank test was applied to continuous variables, McNemar test was used for two-category categorical variables, and Stuart-Maxwell test was performed for any categorical variable having more than two categories. The 10-year cumulative incidence of diverticular disease in both cohorts, with and without appendicitis, was further examined using Kaplan–Meier curves. A comparison of these curves, separately calculated for women and men, was analyzed with the log rank test. Univariable Cox regression analysis was further conducted to determine associations between appendicitis and diverticular disease, with the Cox regression model results expressed as hazard ratios (HRs) with 95% confidence intervals (CIs). Separate Cox regression analyses were performed for different age groups and for women and men. A *p*-value of < 0.05 was considered statistically significant. All analyses were performed with SAS version 9.4 (SAS Institute, Cary, USA).

The study adhered to the recommendations of the Strengthening the Reporting of Observational Studies in Epidemiology (STROBE) guidelines for reporting observational cohort studies.

## Results

### Basic characteristics of the study sample

After propensity score matching, we were able to analyze 25,379 individuals with appendicitis and 25,379 individuals without appendicitis. Table 1 summarizes the baseline characteristics of the study patients. The mean age of the patients with appendicitis was 41.3 years and that of the patients without appendicitis was 41.5 years. There were no significant differences in gender distribution and comorbidities between the two groups. Patients visited a doctor on average 6.0 times per year during the observation period. Importantly, we observed an increase in the incidence of diverticular disease with age in our cohort (Table 2).

**Table 1:** Baseline characteristics of the study sample (after 1:1 matching)

**Variable**	**Proportion among appendicitis patients (*N*, %)** ***N* = 25,379**	**Proportion among non-appendicitis patients (*N*, %)** ***N* = 25,379**	***p*-value**
Age (mean, SD)	41.3 (18.0)	41.5 (18.2)	0.214
Age 18–30	9247 (36.4)	9162 (36.1)	0.488
Age 31–40	4382 (17.3)	4342 (17.1)	
Age 41–50	3934 (15.5)	3927 (15.5)	
Age 51–60	3665 (14.0)	3542 (14.0)	
Age > 60	4251 (16.8)	4406 (17.3)	
Female	14,160 (55.8)	13,999 (55.2)	0.151
Male	11,219 (44.2)	11,380 (44.8)	
Number of physician visits per year during the follow-up (mean, SD)	6.0 (3.8)	6.0 (3.8)	0.918
Charlson Comorbidity Score (CCS)	1.2 (1.7)	1.2 (1.7)	0.823
CCS 0	11,054 (43.6)	11,103 (43.8)	0.596
CCS 1	7575 (29.8)	7567 (29,8)	
CCS 2	3124 (12.3)	3025 (11.9)	
CCS 3	1587 (6.3)	1579 (6.2)	
CCS > 3	2039 (8.0)	2105 (8.3)	

Proportions of patients in *N*, % given, unless otherwise indicated, *SD* standard deviation

**Table 2:** Association between appendicitis and subsequent diverticular disease diagnosis in patients followed in general practices in Germany (univariable Cox regression models)

**Outcome diagnosis**	**Incidence in cases per 1000 patient-years among individuals with appendicitis**	**Incidence in cases per 1000 patient-years among individuals without appendicitis**	**HR for appendicitis (95% CI)**	***p*-value**
Total	6.4	3.5	1.76 (1.57–1.97)	< 0.001
Age 18–30	1.8	1.0	1.75 (1.22–2.51)	0.003
Age 31–40	3.6	2.0	1.76 (1.21–2.56)	0.003
Age 41–50	7.2	3.5	2.03 (1.55–2.65)	< 0.001
Age 51–60	11.6	6.3	1.82 (1.45–2.27)	< 0.001
Age > 60	13.6	8.1	1.67 (1.38–2.01)	< 0.001
Female	6.0	3.7	1.58 (1.36–1.84)	< 0.001
Male	6.9	3.4	2.00 (1.68–2.37)	< 0.001

### Association of appendicitis with subsequent diverticular disease

**Fig. 2:**
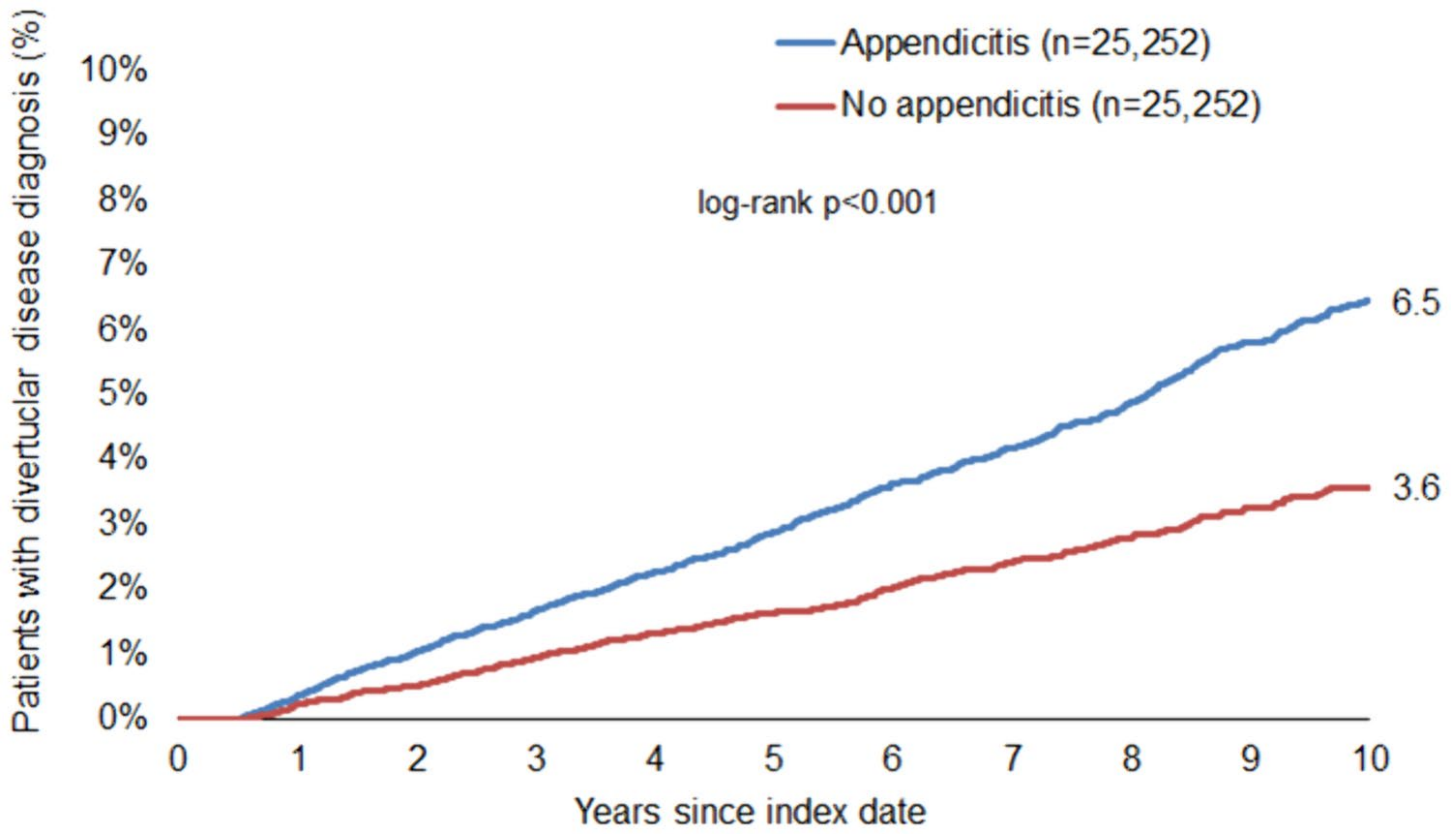
Cumulative incidence of diverticular disease in all patients with and without appendicitis

**Fig. 3:**
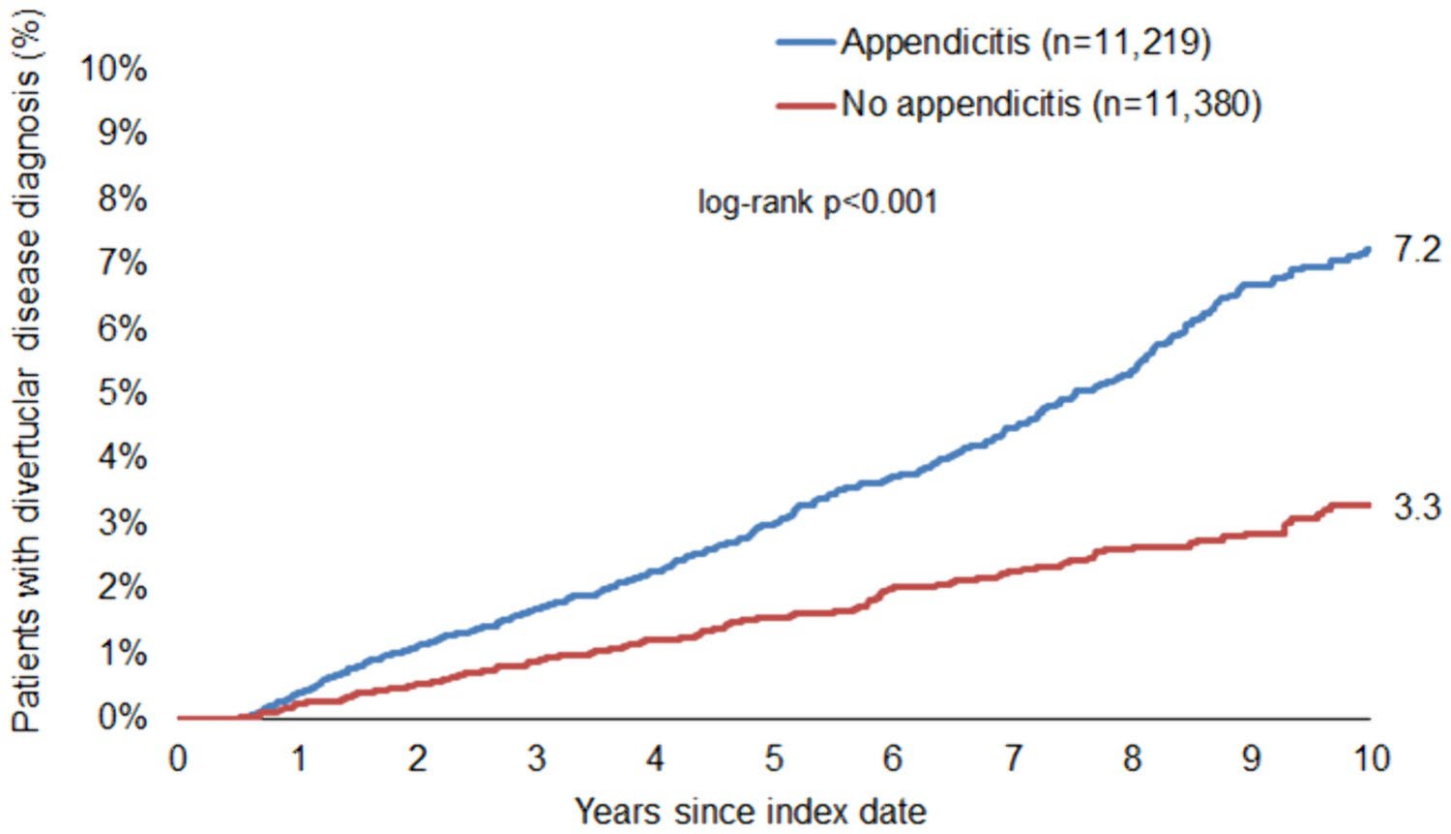
Cumulative incidence of diverticular disease in men with and without appendicitis

**Fig. 4:**
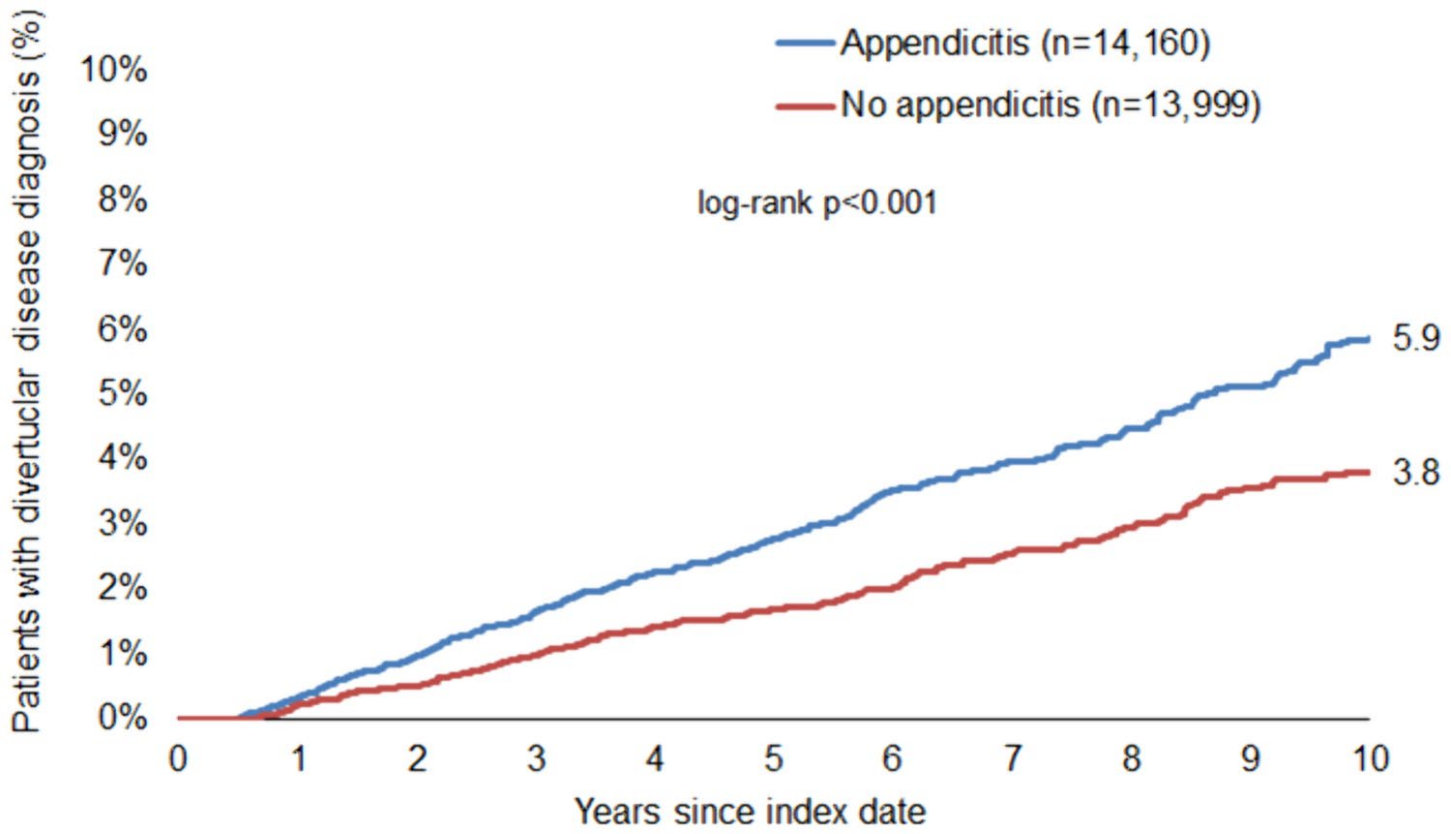
Cumulative incidence of diverticular disease in women with and without appendicitis

First, we examined the incidence of diverticular disease during a 10-year follow-up period separately in patients with a documented history of appendicitis and in the group of patients without appendicitis. Of note, in contrast to 6.5% of patients with appendicitis, only 3.6% of subjects without appendicitis developed diverticular disease over a follow-up period of up to 10 years (Fig. 2). Interestingly, men had a higher cumulative incidence of diverticular disease than women (Figs. 3 and 4).

Next, we performed Cox regression analysis, which confirmed our findings above, significantly associating appendicitis with future diverticular disease in the overall population (HR: 1.76; 95% CI: 1.57–1.97) (Table 2). This association was also significant in each of the five age groups examined. To investigate whether this difference was gender-specific, the regression analysis was also performed in the subgroup of women and men. Interestingly, in this analysis, a history of appendicitis was more strongly associated with future diverticular disease in men (HR: 2.00; 95% CI: 1.68–2.37) than in women (HR: 1.58; 95% CI: 1.36–1.84) (Table 2).

## Discussion

Herein, we present one of the few population-based cohort studies to investigate the link between appendicitis and diverticular disease in a large real-world outpatient cohort in Germany. Using the Disease Analyzer database of more than 1000 primary care physicians in Germany, more than 25,000 adult patients with diagnosed appendicitis were assessed for the incidence of diverticular disease and compared with an identical number of patients without a history of appendicitis who were matched for age, sex, index year, comorbidities, and average number of annual office visits. Overall, the cumulative 10-year incidence of diverticular disease was 3.6% in patients without appendicitis and, remarkably, 6.5% in patients with appendicitis. The regression analysis confirmed a significant association between appendicitis and subsequent diverticular disease in the overall population in each of the five age groups. Interestingly, there were also sex-specific differences, with the association being stronger in men than in women.

As described in the literature, this study showed that the incidence of diverticular disease is clearly age-dependent and increases with age [23]. However, as we have confirmed in our data, it is important to note that diverticular disease can occur at any age. Patients with diverticular disease are predominantly > 40 years of age, but there is evidence that diverticular disease has increased significantly in younger patients (18–44 years), although the reason is not yet clear. It is therefore recommended that diverticular disease should also be included in the differential diagnosis of abdominal pain in younger patients (< 40 years) [24]. In a previous study that analyzed 2127 individuals for the incidence of diverticular disease, the cumulative risk of developing diverticular disease over approximately 10 years (4.3%) was similar to that observed in our study. In contrast to our data, where the incidence increases steadily with age, the authors observed the highest incidence in 40-year-olds (11%), while the incidence decreased by 24% per decade with increasing age [25]. There are differences reported in the literature regarding gender predisposition to diverticular disease [26, 27]. While early studies reported an excess of men among patients with diverticular disease [23], US studies found a 60.7% proportion of women hospitalized for diverticular disease [23, 28].

Similar to our study, there is a nationwide population-based case–control study from Sweden conducted by Sköldberg et al. [14]. The authors examined 41,988 individuals hospitalized with a first diagnosis of diverticular disease between 2000 and 2010, and 413,115 matched controls to determine whether appendectomy with or without appendicitis was associated with an altered risk of hospitalization for diverticular disease. Their results demonstrated that appendectomy, both with and without a diagnosis of acute appendicitis, was associated with an increased incidence of hospitalization for diverticular disease at ages 40–59 and 60–79 years that persisted ≥ 20 years after appendectomy. Thus, appendectomy was present in 6.7% of cases compared to 4.6% of controls. Surprisingly, the strongest association was found for appendectomy within the year before diagnosis of diverticular disease, which the authors attributed to diagnostic uncertainty and errors, such as misdiagnosis of acute diverticulitis as acute appendicitis. At the same time, however, the authors point out that the results for appendectomies performed more than two decades before the diagnosis of diverticular disease suggest that other correlations must exist. However, because Sköldberg et al. found no apparent difference depending on whether acute appendicitis was present at the time of appendectomy, the authors concluded that a predisposition to acute appendicitis, such as common genetic characteristics or environmental factors, is not strongly associated with an increased risk of diverticular disease [14]. Rather, it is hypothesized that appendectomy itself may alter or increase the risk of developing diverticular disease [14].

In addition, two smaller hospital-based case–control studies also found a positive association between appendectomy and diverticular disease, but it should be noted that these studies were methodologically compromised by selection, documentation, and recall bias [12, 13]. For example, an Italian retrospective case–control study included a total of 207 patients, including 57 patients with diverticulitis and 150 patients with asymptomatic diverticulosis, from a colorectal cancer screening program and used a logistic regression function to examine the association between diverticulitis as the dependent variable and several covariates such as sex, age, body mass index, smoking habits, and history of appendectomy. The authors found that independent of smoking, the risk of diverticulitis was up to 4.94 times higher in patients with a history of appendectomy (95% confidence interval 1.98–12.37) than in those without [12].

Similarly, a retrospective cohort study from the USA conducted at a tertiary care center examined the appendectomy rate in a total of 928 patients divided into four patient cohorts (patients with incidental diagnosis of diverticular disease at screening colonoscopy, hospitalized patients with medically treated diverticulitis, patients after left-sided colectomy for diverticulitis, and patients after left-sided colectomy for colorectal cancer) [13]. After adjusting for demographics and other clinical variables, the authors observed that those with diverticular disease were 2.8 times likelier to have had a previous appendectomy compared to the controls (*p* < 0.001). Consequently, the authors proposed similar risk factors and possibly a common pathological link between appendicitis and diverticular disease [13]. However, the hospital-based design of both studies may limit the interpretation of the results, as the study participants may not reflect the general population.

Previous studies have suggested that appendectomy and/or appendicitis are associated with other gastroenterological diseases, such as a lower incidence of ulcerative colitis after appendectomy [8, 29], a significantly risk of developing Crohn’s disease [9] and possibly a higher recurrence rate of Clostridium difficile infections [30].

Changes in the mucosal immune system and intestinal microbiota, as well as effects of the absence of the presumed function of the appendix as a refugium for commensal intestinal microbes, have been proposed as possible biological mechanisms influencing the pathogenesis of other diseases after appendectomy [31], which may also be important for the development of diverticular disease. However, in the study by Sköldberg et al., also, other factors have been discuss that could contribute to the associations shown [14]. These factors could also play a role in our study and possibly contribute to the findings. For example, the experience of having undergone surgery for an abdominal disease may increase the tendency to seek medical help for abdominal complaints, such as diverticular disease, that would have otherwise healed spontaneously. The authors also discuss a tendency to have abdominal pain, which may increase the likelihood of having an appendectomy and receiving a diagnosis of appendicitis, or being hospitalized with a diagnosis of diverticular disease [14].

Interestingly, differences in the gut microbiota between patients with acute appendicitis and healthy individuals have been reported. For example, in a cross-sectional study, Cai et al. examined the association between appendectomy and the composition of the gut microbiota using stool samples from 30 healthy individuals with and without previous appendectomy [32]. The authors concluded that appendectomy affects the composition of the bacterial and fungal gut microbiota. This would lead to the production of short-chain fatty acids (SCFAs), which are thought to play a key role in regulating immunity, protecting the intestinal mucosa, preventing inflammation, and providing energy to epithelial cells. In addition, the appendix has the highest concentration of gut-associated lymphoid tissue (GALT) and is considered important for the immune response of the gastrointestinal tract. Changes in the gut microbiota may have a direct effect on GALT in the appendix, leading to altered immune function [33].

Another study by Shi et al. identified specific bacteria in the gut microbiota after appendectomy that may be involved in intestinal inflammation via induction of cellular immune responses, including induction of leukocyte chemotaxis and stimulation of proinflammatory cytokine production [34]. In addition, there is evidence that Clostridia are more abundant in individuals with acute appendicitis than in healthy controls [33].

Of interest, an association with changes in the gut microbiota has also been described for diverticular disease, with dysbiosis in particular being associated with progression of diverticular disease [35].

Against this background, probiotics are discussed in the literature as a promising adjunctive therapy for the management of acute appendicitis, which should restore the microbial balance, improve the intestinal barrier function, and strengthen the immune function [36]. Probiotics have been shown to reduce postoperative complications and improve postoperative recovery. Petruzziello et al. also provided evidence for the efficacy of probiotics in the treatment of acute uncomplicated diverticulitis (UCD) and found that probiotics contributed to a reduction in inflammatory markers in the blood and stool compared to the placebo group [37]. Ultimately, however, more research is needed to determine the probiotic strains, dosages, and treatment protocols specific to acute appendicitis and the long-term effects.

Finally, a major strength of the present study is the large sample size. In fact, our study is one of the largest population-based studies on this topic and the only one to date that specifically examines the outpatient sector.

In addition, the cohort without appendicitis was matched to the control cohort for age, sex, index year, and even average annual number of consultations. In particular, adjustment for consultation frequency reduces the influence of confounding factors such as utilization patterns. However, we have to admit that there are some limitations caused by our study design. As diagnoses are based on ICD codes, it cannot be excluded that misclassifications or misjudgments may have occurred. For example, the current ICD system is not optimal for distinguishing between different forms of diverticular disease. In addition, it is not possible to extract surgical and procedure codes from the present database, so no conclusions can be drawn about the performance of an appendectomy. However, it should be noted that in Germany surgery is still the gold standard for the management of acute appendicitis, regardless of age, while conservative treatment of acute uncomplicated appendicitis with antibiotics is still the exception rather than the rule. Therefore, the number of cases of appendicitis should be approximately the same as the number of appendectomies. Our study is further limited by the fact that we did not have detailed information on clinical parameters such as medical history, symptoms, clinical examination, laboratory, imaging, and clinical follow-up. Thus, we are unable to provide detailed information on how the diagnoses of the two conditions were made and what diagnostic modalities were used. Similarly, our data do not provide information on the severity of appendicitis (e.g., complicated or uncomplicated) and diverticular disease (e.g., according to the Classification of Diverticular Disease (CDD) [38] or Hinchey classification [39]). There is also no detailed information on some other risk parameters that are associated with diverticular disease and could be potential confounders (e.g., physical activity, diet, nicotine abuse, medications, family, and social history). In addition, it is possible that cases in which a more severe course of diverticular disease was treated in the hospital were missed. However, this study adds to the literature as other authors have only studied inpatients and the outpatient setting has not yet been studied [14].

## Conclusion

Taken together, our results suggest that appendicitis, which often requires appendectomy, may be associated with the risk for developing diverticular disease irrespective of age and sex. The extent to which impairment of the intestinal immune system or microbiome contributes to or explains this association, or whether other causes underlie this association, cannot be determined. Further research is required to further support our study.

## Supplementary Information

Below is the link to the electronic supplementary material.

Supplementary file1 (DOCX 33 KB)

## Data Availability

The data that support the findings of this study are available on request from the corresponding author on reasonable request.
